# Start-Up of a Biofilter in a Full-Scale Groundwater Treatment Plant for Iron and Manganese Removal

**DOI:** 10.3390/ijerph16050698

**Published:** 2019-02-27

**Authors:** Huiping Zeng, Can Yin, Jie Zhang, Dong Li

**Affiliations:** 1 Key Laboratory of Water Science and Water Environment Recovery Engineering, Beijing University of Technology, Beijing 100124, China; 18811715867@163.com (C.Y.); 6282031@163.com (J.Z.); 2 State Key Laboratory of Urban Water Resource and Environment, Harbin Institute of Technology, Harbin 150090, China

**Keywords:** groundwater, iron and manganese removal, biofilter

## Abstract

In recent years, biological purification technology has been widely developed in the process of iron and manganese removal from groundwater. The cultivation and maturation of the biological filter layer are key for biological iron and manganese removal processes. The time needed for maturation varies significantly with the water quality, filter and filter media conditions and operation parameters; sometimes it takes only one or two months, sometime more than half a year. In this paper, the feasibility of adopting an intermittent operation for the cultivation of biofilter was investigated with productive filters in a groundwater treatment plant, and the comparative test of the filter column was conducted. The results showed that the intermittent operation had little effect on the cultivation of the biofilter because dissolved oxygen would be gradually exhausted during the filter-suspension process, making the filter layer anaerobic, thus possibly inhibiting the growth and reproduction of IMOB (Iron and Manganese Oxidizing Bacteria). At the same time, the test shows that when the mature biological filter needs the suspension operation, the emptying method should be considered to avoid the destruction of the biological layer.

## 1. Introduction

Groundwater usually contains iron and manganese in Northeast China, above the allowed maximum concentration levels 0.3 mg/L and 0.1 mg/L for iron and manganese, respectively, according to the Chinese Standards for Drinking Water Quality [1]. Excessive iron and manganese can affect human health and industry production [2,3]. Together with dissolved iron, manganese could form sediments in drinking water distribution lines and incidents of “black” or “brown” water have occurred with the fluctuation of the water supply.

Many methods have been developed since the end of 19th century, mainly divided into chemical oxidation and biological oxidation. Chemical oxidation is usually adopted by traditional water treatment plants [4,5,6,7], including oxidation with atmospheric oxygen assisted by aeration and oxidation by chemical agents such as potassium permanganate, chlorine, chlorine dioxide and ozone. Fe (II) can be oxidized by oxygen at natural pH easily, while the condition of manganese oxidation is stringent. Excessive aeration is often needed to raise the pH to 9–10 to achieve the abiotic oxidation of soluble Mn (II) to insoluble Mn (III/IV) (oxyhydr)oxides. At natural pH, manganese can only be oxidized by the stronger oxidants: ozone, chlorine dioxide, chlorine and potassium permanganate. However, adverse effects could be caused with excessive oxidants. Excessive potassium permanganate will make the color of the filtrate pink. Excessive chlorine dioxide can generate chloric organics such as chlorobenzene, and chlorophenol which are thought to be carcinogens [8,9,10]. 

Biological iron and manganese removal has shown much promise as an effective low-cost way to treat iron- and manganese-contaminated groundwater, becoming more and more common [11,12,13,14] in Europe, Asia, and North America. It was noted that biotic methods could substantially increase treatment capacity and reduce the cost of operation compared with abiotic methods [15]. However, the long time start-up period of the biofilters is a main drawback for their popularization and application. Chemical oxidation of iron and manganese occurs in several minutes or even several seconds by strong oxidants. Therefore, almost no time is needed for the start-up of a water treatment plant for iron and manganese removal adopting the abiotic oxidation process. For the plants adopting the biotic oxidation process, 1–2 months or even more time is needed for the maturation of the biofilter [16,17] because time is needed for the attachment of inoculated bacteria to the filter media, and time is needed for the acclimation and enrichment of functional bacteria. 

Recently, several biological groundwater treatment plants have been built in Northeast China, which have been operated successfully and the filtrate quality is better than the Chinese Standard, but because of the difference in water quality, filter material and operation parameters, the start-up time varies from one month to eight months [18,19,20,21,22]. 

The establishment of a biological layer is the first step for the start-up of the biological iron and manganese removal process. The efficient and stable ability of iron and manganese removal depends on the establishment of a stable and complex ecosystem with iron- and manganese-oxidizing bacteria as dominant bacteria [23], but these bacteria are microorganisms with a low metabolic rate and growth rate. Therefore, the biofilter for iron and manganese removal is very different from the biofilter for carbon and nitrogen removal.

So far, there are not many references on the start-up of the biological iron and manganese removal filter except that when ammonia nitrogen is contained in raw water, the removal of manganese requires complete nitrification of ammonia nitrogen, so the maturation of the filter takes 3–4 months [24,25].

This paper provides a specific example of the start-up of biological groundwater treatment removal for iron and manganese removal. Because of the external pipe network problems, the water plant can only be operated intermittently. Therefore, the effect of the cultivation of an intermittent water supply biological filter layer was analyzed with the aid of a filter column test, in order to provide more references and accumulate more experience for the start-up of the biological iron and manganese removal filter layer.

## 2. Materials and Methods

### 2.1. Water Treatment Plant and Groundwater

**Figure 1:**
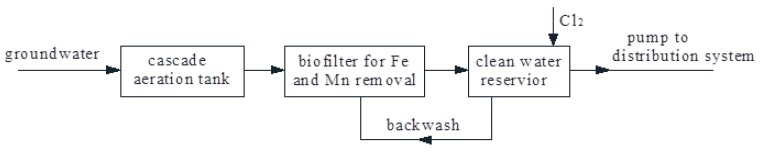
Flow chart of Water Treatment Plants.

Shenyang is the capital of Liaoning Province in Northeast China. Groundwater is an important water supply source in Shenyang. The groundwater temperature is 10–12 °C all year round due to the latitude. In this paper, the groundwater came from 16 deep wells (depth 60–100 m). The water outputs were between 100 to 150 m^3^/h; the groundwater characteristics are shown in Table 1. The total treatment capacity of the WTP (Water Treatment Plant) is 3 × 10^4^ m^3^/d; the flow chart is shown in Figure 1, with the aim that the simultaneous removal of iron and manganese occurs in one filter. After the raw water was extracted from the ground, it first entered the cascade aeration tank for oxygen filling, which provided sufficient oxygen for the subsequent bio-oxidation of iron and manganese in the biochemical filter, where iron and manganese ions were oxidized into solid iron and manganese oxides, which were intercepted and removed by the filter layer. Finally, the purified water entered into the clear water reservoir and then the distribution pipe network. With the extension of filtration time, the interception impurities of the filter layer increased, and the filtration resistance increased sharply, and the filter backwashing process was needed. The filter had a depth of 1000 mm, using sand as the filter media, with a diameter of 0.5–1.2 mm. 

Raw water from waterworks was basically used as the influent for the filter column test, and chemical agents can be added to change the content of iron, manganese and other substances according to the need.

### 2.2. Start-Up Process

Due to the construction of the external pipeline network, water plants could only be operated intermittently at the initial stage of biofilter cultivation, and then gradually transit to continuous operation. The whole operation process is shown in Table 2.

### 2.3. Filter Column Test

**Figure 2:**
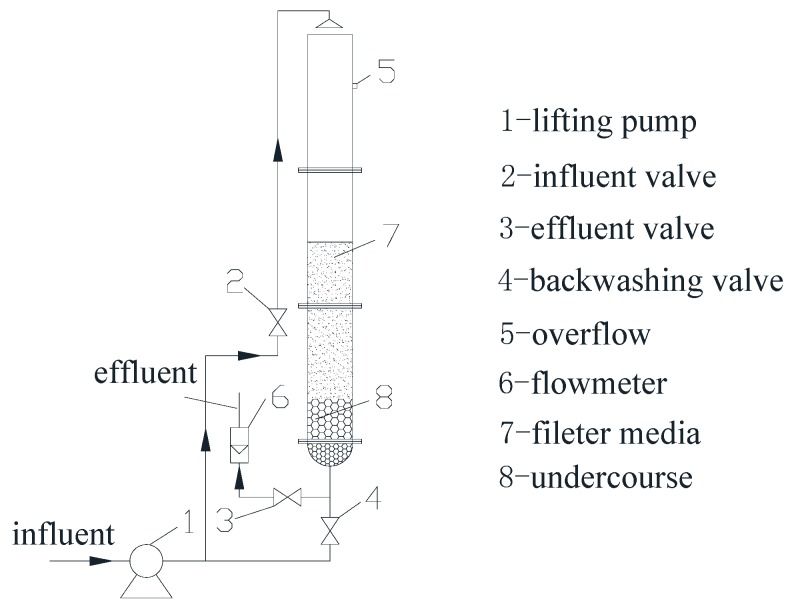
Flow chart of the filter column.

The test device is shown in Figure 2. The filter column was made of Plexiglas with a height of 3 m and an inner diameter of 250 mm. A sampling port was set every 10 cm along the direction of the filter layer. In order to simulate the actual productive filter, the filling of filter media and the inoculation amount were consistent with those in filter 1#. The filter column adopted a downward flow, and the filter speed was controlled by a flowmeter to ensure uniform filtration. The backwashing strength was controlled by the expansion rate of filter media, using about 15–20%. The influent and backwash water were both from the aeration tank.

### 2.4. Analysis Methods

The influent was taken from the reserved sampling port of the inlet pipe of the cascade aeration tank and the effluent was taken from the outlet pipe of the biofilter. All samples were analyzed immediately in the laboratory of the Water Treatment Plant, so no storage measures were adopted. All iron and manganese analyses were performed by a spectrophotometer in accordance with standard methods [26]. Dissolved oxygen was measured by a Dissolved Oxygen Meter (oxi340i, WTW, Munich Germany), pH was determined using an electrode (pH/oxi340i, WTW, Munich Germany)

### 2.5. Filter Inoculation

Source of bacteria: From the engineering practice of a biological iron and manganese removal water treatment plant, it was found that autochthonous IMOB were contained in groundwater containing iron and manganese all over different regions, and they were adaptable for the local water environment. In the process of biofilter establishment, the local bacteria was added to the filter after amplification and cultivation, which showed strong adaptability and promoted the maturity of the biofilter as soon as possible and greatly shorten the cultivation period [23]. Therefore, in this study, the ferromanganese oxidizing bacteria in the mud of the aeration tank wall of the local groundwater treatment plant for iron and manganese removal were collected and cultured, and then inoculated into the filter.

Methods: A high concentration of bacterial liquid was used to inoculate the filter at one time, and a large number of bacteria were brought into the filter through some operation methods, then the filter was cultured at a low filtration rate. Under the condition of no medium and low nutrients, the bacteria were directly cultured by raw water. The bacteria proliferated slowly in the initial stage of culture, it was beneficial to further expand and cultivate the biological population suitable for the characteristics of raw water in the filter layer [23]. In order to study the effect of inoculation amount on the start-up of biofilter, filter 3# was selected to inoculate twice the amount of bacterial liquid among these six filters. When the effluent was not up to the standard, the biological filter was considered to be mature. 

## 3. Results and Discussion

### 3.1. Full-Scale Filter Test

#### 3.1.1. Initial Intermittent Operation Stage

**Figure 3:**
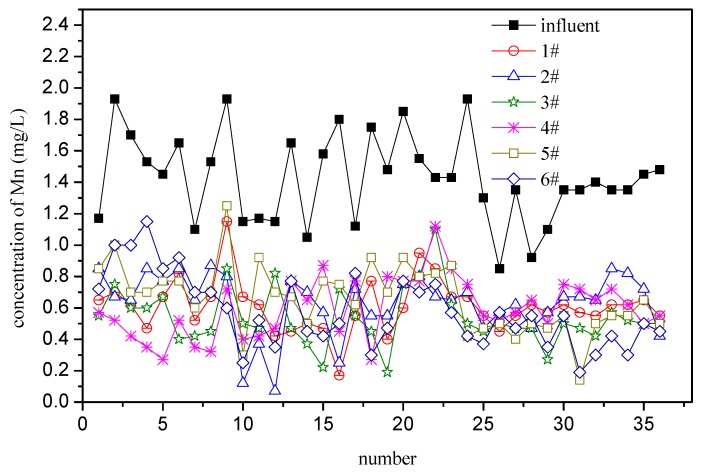
Mn in the influent and effluent (during the stage of intermittent operation; samples are taken once every three days of operation).

The results are shown in Figure 3. The filters had a certain manganese removal capacity due to the inoculation at the initial stage of operation, and the manganese removal rate was about 50%; there were some differences between these 6 filters due to the initial difference of the filters and the difference of operation which could not be completely the same, but the trend was similar. After five months of operation, the manganese concentration in the effluent of the filter did not show a downward trend with time, it just fluctuated up and down, indicating that the number of ferromanganese oxidizing bacteria in the filter has not been proliferated and the activity was in an unstable stage [23].

#### 3.1.2. Continuous Operation Stage

**Figure 4:**
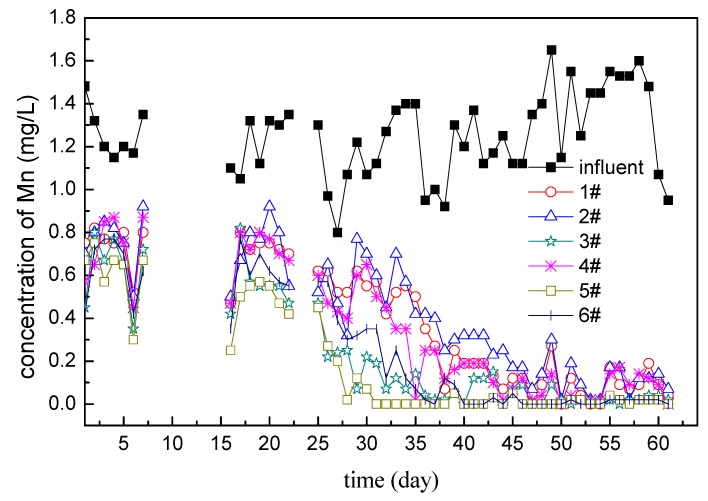
Mn in the influent and effluent (during the stage of continuous operation; samples are taken once every day of operation).

The effluent quality of the filters showed in Figure 4 has been significantly improved in the continuous operation stage, but was not the same as the intermittent operation stage. The effluent of filter 3#, 5#, 6# came close to the standard after one month of continuous operation, and tended to reach the standard steadily in the next one month. Also, the effluent of filter 1#, 2#, 4# came close to the standard after 50 days of continuous operation, and then it took about 10 days to reach the standard. But at that time, the filters were not fully mature, so the effluent of filter1#, 2#, 4# fluctuated slightly.

Generally, after inoculation, microorganisms will undergo a period of adaptation under stable and suitable conditions so as to achieve a logarithmic growth period of rapid growth and rapid propagation. In this stage, the quality of effluent water will quickly improve with the mass propagation of microorganisms, and finally achieve the effluent standard [23]. But the appearance of the logarithmic growth phase depends entirely on the operation condition. The actual operation results showed that the intermittent operation in the first five months greatly delayed the microorganisms entering the logarithmic growth period, and thus delayed the maturity of the biological filter. In the subsequent filter column tests, it was verified that under the same conditions, the filter column did achieve maturity of the biological layer in about 40 days after continuous operation.

According to the figure, the continuous operation process could be divided into two stages. The first month was a period of rapid decline of the manganese concentration in the effluent, and the second month was a period of fluctuation of the effluent in a low manganese concentration. Although the inoculation amount of filter 3# was twice as much as that of the other five filters, and the removal efficiency of filter 3# was better than that of filters 1#, 2#, 4# in the first month. However, filter 6# has almost the same effect as filter 3#, and filter 5# did even slightly better; these differences might stem from the fact that it is impossible for the filter and the operation to be completely the same. Finally in the second month, the six filters all entered the same fluctuation stage with a low concentration of manganese, i.e., basically no difference. 

It can be inferred from the experimental results that the maturation of filter layer was not directly related to the amount of inoculation completely because filter 3# was better than 1#, 2#, 4# in the early stage, but 5#, 6# was similar to 3#, and even 6# was better than 3#. Therefore, in the future inoculation process, the appropriate amount is enough, not the more the better.

#### 3.1.3. Speed-Up Stage

**Figure 5:**
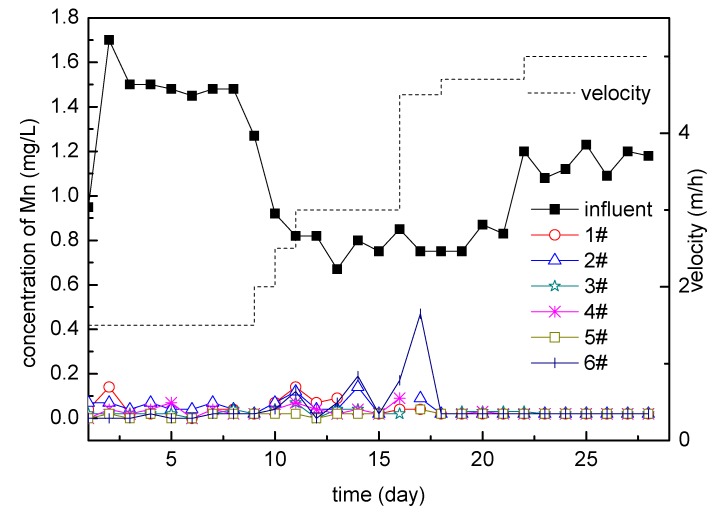
Mn in the influent and effluent (during the stage of speed-up; samples are taken once every day of operation).

In the granular media filtration process, the shear force on the surface of the filter material rises with the increase in flow rate [27]. Therefore, in the beginning of the cultivation, a low filtration rate was usually adopted [28], in case of reducing the shear force to promote the adhesion and fixation of bacteria, which were in the mud or free in the water, onto the surface of the filter media. Therefore, 1.5 m/h was the initial filtration rate for the culture of biofilter in this plant, and the speed-up was carried out after the effluent of the filter reached the standard for one week. In the beginning, the increase for the speed-up was only 0.5 m/h. After the effluent reached the standard, the next speed-up started, and the filtration rate ranged from 1.5 m/h to 2 m/h to 2.5 m/h and then to 3 m/h. In this process (Figure 5), the effluent quality of individual filters fluctuated slightly for a short time, but soon the effluent quality recovered. Therefore, the speed-up was accelerated from 3 m/h to 4.5 m/h to 4.7 m/h and then to 5 m/h; in the first two days, the effluent of 6# fluctuated, but on the third day, it recovered to below the standard again. This indicates that the biological filter has been fully cultivated.

### 3.2. Pilot-ScaleFilter Column Test

#### 3.2.1. Start-Up of the Filter Column

**Figure 6:**
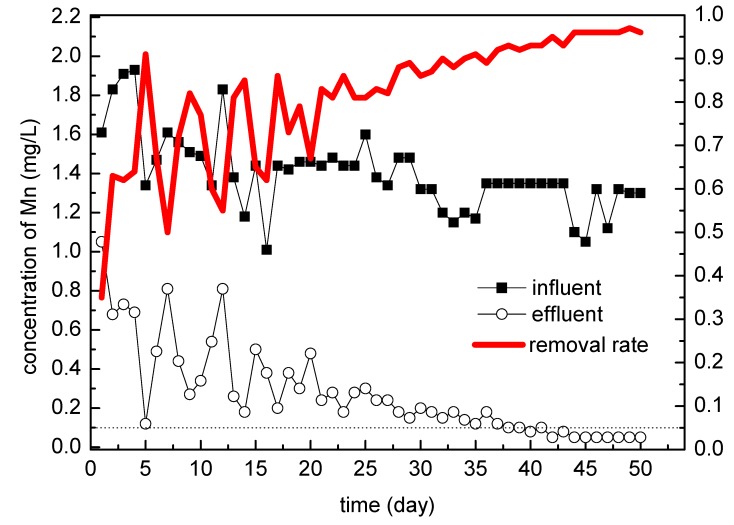
Mn removal and rate of the filter column.

The concentration of manganese in the effluent in Figure 6 fluctuated around 0.6 mg/L after the operation of the filter column, which is similar to the actual production filter, but the concentration of manganese in the effluent decreased gradually, and was finally below 0.1 mg/L within 40 days; the removal rate was as high as 95%, and all these phenomena indicate that the biological filter layer was mature. It can be found that the manganese variation curve of the effluent from the filter column was similar to that of the effluent from the production filter after continuous operation. Therefore, in the first four months of intermittent operation of the water plant, the cultivation of the biological filter almost had no effect. The reason could be that the intermittent operation could not provide a good environment for the microorganisms in the filter layer, so the microorganisms have been staying in the adaptive stage and have not entered the logarithmic growth stage [23].

#### 3.2.2. Suspension and Rerun of the Filter Column

As has been noted, the intermittent operation had little effect on the cultivation of the biofilter. In order to find the difference between intermittent operation and continuous operation, the suspension and rerun tests of filter column were carried out, under the water quality of low iron and manganese and high iron and manganese with micro-pollution.

(1) Groundwater quality of low concentrations of iron and manganese

**Figure 7:**
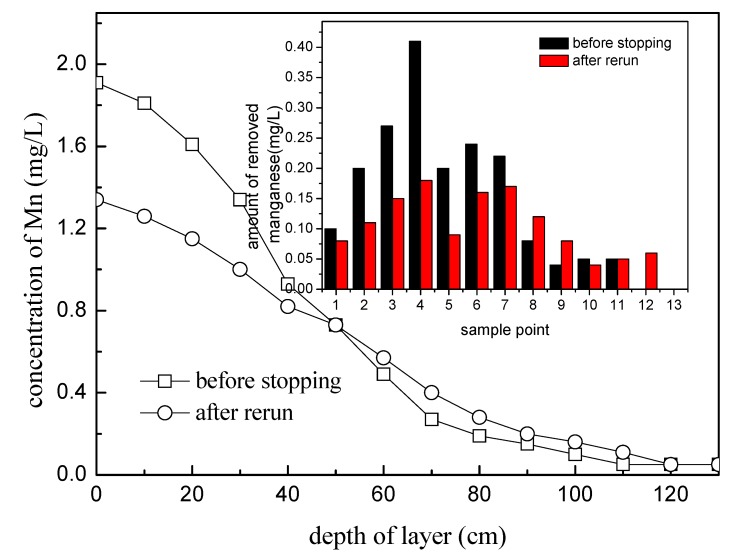
Mn concentration at the different height layers of the filter column before stopping and after rerun.

After several days of stopping operation, the biofilter column restarted, and the manganese removal capacity of the filter column decreased significantly (Figure 7). The manganese removal capacity of the first 50 cm filter layer decreased from 1.18 mg/L to 0.61 mg/L, only 50%. At the same time, it was found that the filter column was in an anoxic state with a DO of zero. Without the basic need of oxygen for the manganese oxidizing bacteria, their functions would decrease [29], so the manganese removal rate had a sharp decrease.

(2) Groundwater quality with a high concentration of iron and manganese

**Figure 8:**
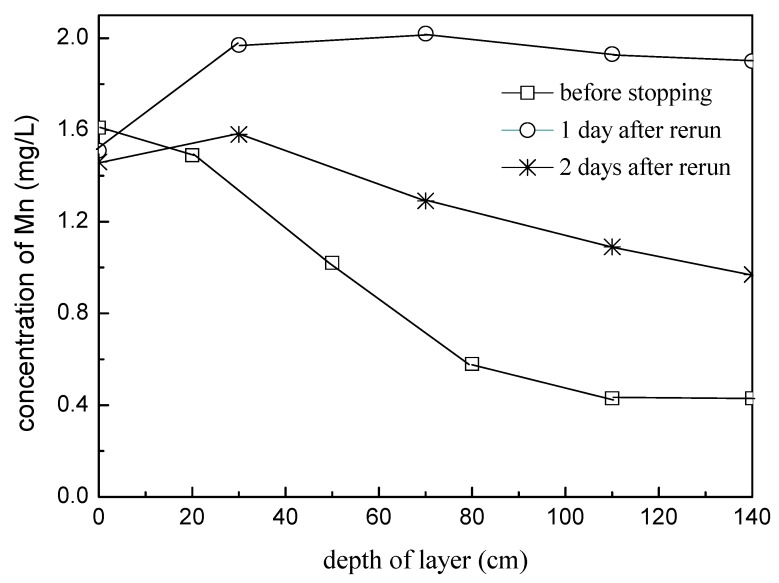
Mn concentration in the filtrate along filter depth (soaked state).

Two biofilter columns were used for the suspension and rerun test; during the suspension stage, one column was kept empty, and the other was full of raw water. Two distinct phenomena appeared as shown in the figure, when the two columns restarted to operate. For the column kept full of raw water (Figure 8), the manganese content in the effluent exceeded that of the influent, and it recovered slightly the next day, costing four days for the completely recovery (the data are not shown). The results showed that the dissolution of manganese occurred during the suspension process, while it was found that the dissolved oxygen in the water was zero. This two phenomena could be explained that due to the suspension of filter column with the situation of full of water, there was no way for the supplementation of oxygen for the filter layer. Therefore, when the dissolved oxygen in the layer was gradually depleted by reducing substances, it became anaerobic, which led to the dissolution of high valence manganese (Mn^4+^), and the concentration of manganese in the effluent was higher than that in the influent. This phenomenon is extremely harmful to the cultivation of the biofilter layer, because most of the Manganese Oxidizing microorganisms construct their living space with manganese oxides batched on the filter material [23]. With the dissolution of manganese oxides, the biofilm of the filter layer will be greatly destroyed. Its recovery will also take a long time.

**Figure 9:**
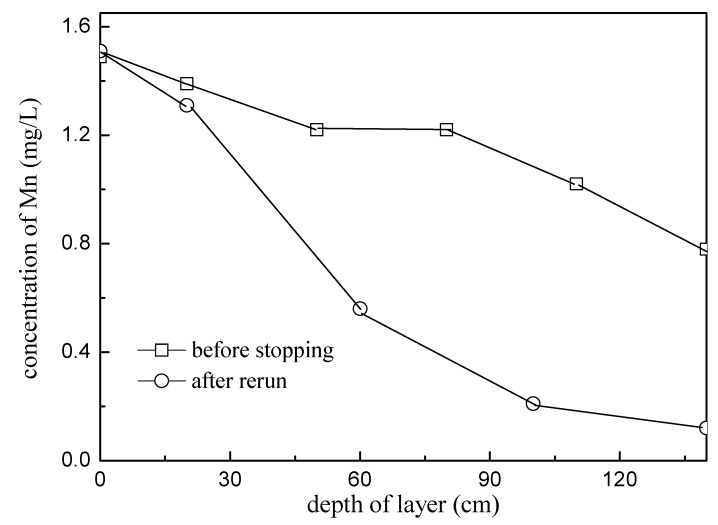
Mn concentration in the filtrate along filter depth (empty state).

The air in the filter column kept empty (Figure 9) was in a circulation state, which made the filter column an aerobic environment, and provided oxygen for the microorganisms. Yet due to no supply of raw water, the microorganisms in the filter column stayed in a long-term starvation state. So when the filter restarted, the manganese in the raw water was utilized by microorganisms in large quantities, which led to the phenomenon of low manganese in the effluent water at the initial stage of the rerun operation of the filter column. After a few days of operation, the concentration of manganese in the effluent returned to its original state (data not given).

(3) Reflections of the test of Suspension and Rerun 

Generally, substrate and oxygen are two basic demands for the cultivation of microorganisms in the biological manganese removal filters. The shutdown process of filter without emptying will deplete the residual dissolved oxygen in the filter layer, which will eventually lead to the anaerobic state, thus affecting the activity and metabolism of microorganisms. Especially when there are some reducing substances in raw water, anaerobic conditions may cause the re-dissolution of manganese oxides which are an important structural component of biofilm, finally causing serious damage to the manganese removal biofilter. Therefore, it is suggested that in the process of cultivation of the biological filter or operation of mature filter, if it is necessary to shut down the tank, it should be kept empty, not full of water.

## 4. Conclusions

1. With the initial intermittent operation and the late continuous operation, the plant took 7 months to complete the start-up of biological iron and manganese removal filters.

2. By comparing the production filter and the filter column test, it can be inferred that although the intermittent operation reduced the backwashing frequency, no contribution was made to the cultivation of the biological manganese removal layer.

3. For the stopping operation of the biological filter in the start-up or stable operation stage, it is suggested to use filter emptying to ensure that the filter can be in an aerobic state, which is beneficial to the aerobic microorganisms and the biofilter layer.

## Figures and Tables

**Table 1:** Characteristics of the groundwater.

Parameter	Value	Parameter	Value
Temperature (°C)	12–14	Total dissolved solids (mg/L)	240–260
pH	6.7–6.9	DO (mg/L)	0
Turbidity (NTU)	32.3–39.0	Conductivity (Us/cm)	268–280
Mn^2+^ (mg/L)	0.8–2.0	Fe^2+^ (mg/L)	0.15–0.20

**Table 2:** Operation process during the biofilter cultivation.

Stage	Frequency of Operation	Velocity	Time
Intermittent Operation	Once three days, 6 h at a time	1.5 m/h	Two months
	Once a day, 7 h at a time	1.5 m/h	Two months
Continuous operation	Continuous running	1.5 m/h	Two months
speed-up	Continuous running	1.5 m/h to 5 m/h step by step	One month
